# New European rules introduced by regulation (EU) 2016/429 to facilitate animal trade: With great risk comes great responsibility

**DOI:** 10.3389/fvets.2022.1003732

**Published:** 2022-10-19

**Authors:** Guido Ruggero Loria, Sergio Migliore, Carmelo Bongiorno, Gabriele Ciaccio, Alberto Laddomada

**Affiliations:** ^1^Area Diagnostica Specialistica, Istituto Zooprofilattico Sperimentale della Sicilia “A. Mirri”, Palermo, Italy; ^2^Consultant, Cagliari, Italy

**Keywords:** animal health, animal trade, animal movement, animal welfare, EU regulation, Animal Health Law

## Introduction

In the 1960s, the six founding countries (Belgium, France, Italy, Luxembourg, the Netherlands, and West Germany) of the European Economic Community (EEC) initiated a process for the harmonization of livestock trade and its products among Member States (MSs) and third countries. In those years, several major animal diseases (vesicular diseases, such as foot-and-mouth disease, classical swine fever, and contagious bovine pleuropneumonia) were widespread in Europe, with severe economic implications. To share a common strategy, the EEC promoted a harmonization process of the regulatory differences between the countries, with the aim of establishing one single market for live animals and their products.

This process has been ongoing for the last 50 years, with continuous revision and updating of the wide body of rules on the legislation on animal health and food safety. This has gradually harmonized the legal framework in the European Union (EU), which currently includes 27 Member States and approximately 450 million citizens (1). This ambitious project has been successful, and all EU citizens have taken major advantage of the single market, for example, in terms of the availability of animal proteins at reasonable prices.

In 2016, a further ambitious step forward has been taken by means of Regulation EU 2016/429 (so-called “Animal Health Law—AHL”) (2), which has been adopted to establish a single regulatory tool to cover all aspects of animal health.

The new approach of AHL by the application of proactive measures should facilitate trade within the boundaries of the EU (internal trade), minimize border controls and bureaucratic procedures between EU MSs, and eliminate the crowd of bilateral agreements applied so far. Moreover, the new system must guarantee and accelerate the traceability and sharing of data on the identification and certification of the moved animals to be ready for early detection and rapid warning of disease risk.

This policy facilitates animal movements by more stringent biosafety guarantees, which are required for those countries of dispatch. Furthermore, introduced innovations have over time attributed ever greater responsibilities to all relevant actors involved in all steps of the animal production chain.

To better understand the evolution of this regulation, we went through and considered all major steps taken to date.

## Legislative basis

### Animal health and trade

The first European Directive concerning animal movement among MS (Council Directive 64/432/EEC dated 24 June 1964) (3) established the conditions for movements within the Union of domestic animal species with greater commercial importance: cattle and pigs. The Directive was specially addressed to limit the risk of spreading some “priority diseases,” establishing that both species could be moved only under the condition of the absence of clinical signs of infectious diseases, and/or if they came from herds declared free from a list of pathogens: Directive 82/894/EEC (4), Directive 92/119/EEC (Annex EI and Part II) (5), and the CD 64/432/EEC (which added other diseases). In the same years, the conditions for the movements of Equidae Directive 90/426/EEC (6) and small ruminants (Directive 91/68/EEC) (7) were also established.

With the strengthening of the Common European Market, further conditions for the movement of animals in the EU were introduced (Directive 90/425/EEC) (8). The MS had to ensure that: (i) farmers and animal-origin products were obliged to respect national and European regulations. (ii) their animals and animal-origin products intended for intra-community trade must be controlled as if they were destined for the internal market of own country, and (iii) the means of transport must guarantee high levels of biosecurity. It was an innovative step forward, which replaced the previous strict regime of national border controls between MSs on all consignments of live animals with non-discriminatory inspections, randomly performed at the places of destination.

### Animal welfare and transport

With the issuance of Directive 64/432/EEC (3), higher standards for animal welfare during transportation were introduced. A few years later, Directive 91/628/EEC (9) added proper rules for each species, regarding the duration and number of stops of carriage and registration and authorization of animal transporters. Mandatory appropriate training for transporters was also requested, with more complete records for international transport and related sanctions for non-compliance.

However, only with the issuance of EC Regulation 1/2005 (10), a real step ahead toward more appropriate animal welfare during transport was made, including mandatory traceability of all stages of the journey.

## The new rules

The great part of the AHL (2) is mainly based on rules defined in previously existing Union Acts, adapted to the new legal framework considering experience learnt from the past, updates of international standards, modern epidemiological approaches as well as scientific progress, World Organization for Animal Health (WOAH) Standards (11), epidemiological data, and European Food Safety Authority (EFSA) opinions. The new approach is a more coherent expression of the “One Health” concept, drafted considering the link between animal and public health, wildlife, environment, food and feed safety, animal welfare, food security, economic aspects, and social and cultural issues. The AHL consists of 283 articles that repeal 38 decisions, directives, and regulations adopted from 1964 up to today. Since the publication of Reg. 2016/429, more than 30 different delegated and implemented acts have been prepared concerning: the list of the diseases subject to notification to competent veterinary authority (Commission Delegated Regulation (CDR) (EU) 2018/16292018/1629—Diseases list) (12); their control measures, which are now not focused anymore on “single” diseases but grouped in five categories according to the risk (Commission Implemented Regulation (CIR) 2018/1882) (13) and their control measures (CDR 2020/687 (14); the rules for animal movements within the EU to and from third countries coming into force by the CDR 2020/688 (15).

A novelty is a new way to consider the risks related to animal diseases: now each pathogen is included in a category according to the intensity of control measures to be applied. It is called “categorization” as regulated by Article 9. Each category includes groups of animal diseases that can affect various susceptible species, grouped by epidemiological characteristics. Therefore, all the 63 diseases listed in the CDR 2018/1629 (Table 1) are grouped into 5 categories (A, B, C, D, and E).

**Table 1:** Annex II—A list of animal diseases of the Animal Health Law (AHL).

Infection with rinderpest virus	Infection with Rift Valley fever virus	Infection with *Brucella abortus, B. melitensis* and *B. suis*
Infection with *Mycobacterium tuberculosis* complex (*M. bovis, M. caprae and M. tuberculosis*)	Infection with rabies virus	Infection with bluetongue virus (serotypes 1–24)
Infestation with *Echinococcus multilocularis*	Infection with epizootic hemorrhagic disease virus	Anthrax
Surra (*Trypanosoma evansi*)	Ebola virus disease	Paratuberculosis
Japanese encephalitis	West Nile fever	Q fever
Infection with lumpy skin disease virus	Infection with *Mycoplasma mycoides* subsp. *mycoides* SC (Contagious bovine pleuropneumonia)	Infectious bovine rhinotracheitis/infectious pustular vulvovaginitis
Bovine viral diarrhea	Bovine genital campylobacteriosis	Trichomonosis
Enzootic bovine leukosis	Sheep pox and goat pox	Infection with peste des petits ruminants virus
Contagious caprine pleuropneumonia	Ovine epididymitis (*Brucella ovis*)	Infection with *Burkholderia mallei* (Glanders)
Infection with equine arteritis virus	Equine infectious anemia	Dourine
Venezuelan equine encephalomyelitis	Contagious equine metritis	Equine encephalomyelitis (Eastern and Western)
Infection with Aujeszky's disease virus	Infection with porcine reproductive and respiratory syndrome virus	Infection with Newcastle disease virus
Avian mycoplasmosis (*Mycoplasma gallisepticum* and *M. meleagridis*)	Infection with *Salmonella Pullorum, S. Gallinarum* and *S. arizonae*	Infection with low pathogenic avian influenza viruses
Avian chlamydiosis	Infestation with *Varroa spp*. (Varroosis)	Infestation with *Aethina tumida* (Small hive beetle)
American foulbrood	Infestation with *Tropilaelaps spp*.	Infection with Batrachochytrium salamandrivorans
Epizootic hematopoietic necrosis	Viral hemorrhagic septicemia	Infectious hematopoietic necrosis
Infection with highly polymorphic region (HPR) deleted infectious salmon anemia virus	Koi herpes virus disease	Infection with *Mikrocytos mackini*
Infection with *Perkinsus marinus*	Infection with *Bonamia ostreae*	Infection with *Bonamia exitiosa*
Infection with *Marteilia refringens*	Infection with Taura syndrome virus	Infection with yellow head virus
Infection with white spot syndrome virus		

For all these diseases, specific restrictions are provided (according to Article 18, paragraph 1), including the immediate notification to the competent authority and, according to an information system regulated by Articles 22 and 23, which will be established by the EC (in the next future, it will replace the current Animal Disease Notification System ruled by Directive 82/894/EEC).

Considering the CIR 2018/1882, with reference to Article 128 of Reg. 429/2016 (listing and categorization of animal diseases), animal production operators will not be able to move animals destined to be culled in another MS if they are part of mandatory or voluntary eradication programs as provided by Article 31, for the diseases of categories referred to in letters a), b), and c) of Article 9. In relation to the diseases mentioned in Article 9 letter d), operators will be able to move livestock to another MS only if they fulfill all health requirements shared among MS with AHL enactment.

Among the health requirements strengthened by AHL, biosecurity certainly represents the principal tool to prevent the risk of pathogen introduction into the food chain. Biosafety has become a strategic prerequisite for efficient health management of farms: farmers, operators, all professionals of the animal production sector must apply the necessary knowledge on biosecurity on the farm, to the means of transport, to assembly centers, to animal markets, and exhibitions. The application of biosecurity measures is under the responsibility of all operators in the animal production sector and is supervised by competent authorities. Consolidating the previous rules in the EU, all movements of terrestrial ungulates must be accompanied by a health certificate as reported in the AHL (Section 7, Article 143) and related CDR 2020/688, Chapter 8, section 1, Articles. 69–72 (which can be digital), *via* the TRAde Control and Export System (TRACES) system.

Operators other than transporters are required to notify (Article 152) the competent authority of their MS of origin the expected movements of animals; the latter is required to notify the competent authority of the state of the movements (Article 153).

The entry into the EU of live animals and their products from third countries is regulated by Articles 229–239, which provide that imports can only take place from countries and establishments that fulfill all necessary guarantees on the health status of their territory, as well as the capacity for surveillance and control of the listed and emerging diseases, considering epidemiological data on previous entries of animals and products at the EU entry points. Upon entry into the EU, animals coming from third Countries are subjected to official controls as stated by Reg. (EU) 2017/625, to ascertain compliance with the provisions on welfare (Article 21) and compliance with animal health legislation (Article 44).

More “preparedness” has been introduced with the AHL: Article 6 clarifies the definition of “emerging diseases”: “a disease which results from the evolution or modification of an existing pathogen, which spreads to an area or to a new host species; identified for the first time in the EU or caused by an unrecognized or previously unrecognized pathogen.”

## Discussion

Thanks to the work and mediation between the 27 Member States and the United Kingdom, the great step toward a European law on animal health has been made coherently with previous experience and current epidemiological risk evaluation approaches.

With the introduction of Reg. 2016/429, some novelties can be remarked: a revised list of 63 diseases has been considered for the certification and traceability of the origin of susceptible species. Some diseases (brucellosis, tuberculosis) are now managed with a different approach involving more pathogens (TB complex) of the genus and more species (multispecies diseases), whereas others (bluetongue) have been reconsidered, approving eradication plans only for those territories which voluntary require such a certification. Moreover, AHL will delete from the list of notifiable diseases, certain pathogens historically included, such as vesicular diseases of swine and horses and Teschen disease.

In addition, much more coherence of AHL is applied between scientific knowledge and the most updated epidemiological achievements with a new, more realistic, approach for those “unpredictable outbreaks” where vectors or migratory species have a primary role in their occurrence. In these cases, the decision of the Commission is limited to implement and strengthen the risk assessment in the EU, avoiding expensive and often ineffective control measures against insects or worse, against viruses carried by migratory birds.

Another new point is the introduction of disease management and surveillance of unusual “exotic” livestock species, such as cervids and camelids, which now require similar rules to those of domestic ungulates.

With the new rules, an infected region, characterized by disease outbreaks in domestic or wild animals, can continue its commercial activity by guaranteeing (under the responsibility of the Veterinary Authority) proper biosafety measures sufficient to prevent the risk and to certify the movement of animals and their products.

The other main advantage of the AHL is its flexibility: according to epidemiological evidence, it allows to issue new Delegated or Implementing Acts (respecting the AHL legal context) to better adapt to the new epidemiological situation or to react to new emerging risks. All these changes, contribute in improving surveillance and control policies for animal health in the EU, modulating veterinary measures to the entity of the risk and to the sustainability of the actions.

In conclusion, the main pillars on which AHL relies are: a renewed, central role of the veterinarians and their full responsibility in the prevention perspective; the requirement of training initiatives; the identification of priority diseases; an improvement of rapid and centralized, communication and information technologies; and the importance of biosecurity management of livestock settlements. Therefore, more professionality and training are required and vets represent the reference professionals who must bring this new EU sanitary policy into the local productive reality.

## Author contributions

GL and AL: conceptualization. GL and SM: writing—original draft preparation. GL, SM, CB, and AL: writing—review and editing. GL, GC, and AL: supervision. All authors have read and agreed to the published version of the manuscript.

## Conflict of interest

The authors declare that the research was conducted in the absence of any commercial or financial relationships that could be construed as a potential conflict of interest.

## Publisher's note

All claims expressed in this article are solely those of the authors and do not necessarily represent those of their affiliated organizations, or those of the publisher, the editors and the reviewers. Any product that may be evaluated in this article, or claim that may be made by its manufacturer, is not guaranteed or endorsed by the publisher.
